# Image-enhanced endoscopy improves visibility of endoscopic images in individuals with color vision deficiency

**DOI:** 10.1055/a-2714-2766

**Published:** 2025-11-06

**Authors:** Akiko Ohno, Jun Miyoshi, Mitsunori Kusuhara, Yoko Jinbo, Ryosuke Kaji, Takahiro Shirakawa, Moegi Watanabe, Shiori Tsubata, Ryutaro Sumi, Minoru Matsuura, Koichi Iga, Masataka Okabe, Tadakazu Hisamatsu

**Affiliations:** 1 12912 Department of Gastroenterology and Hepatology, Kyorin University, Tokyo, Japan; 2 13874 Endoscopy Division, National Cancer Center Japan, Chuo, Japan; 3 Color Universal Design Organization, Tokyo, Japan; 4 12839 Department of Anatomy, Jikei University School of Medicine, Minato, Japan

**Keywords:** Endoscopy Upper GI Tract, Diagnosis and imaging (inc chromoendoscopy, NBI, iSCAN, FICE, CLE), Endoscopy Lower GI Tract, Quality and logistical aspects, Quality management

## Abstract

**Background and study aims:**

Color vision deficiency (CVD) may affect perception of color information in gastrointestinal endoscopy. However, the color-universal concept in endoscopy remains underappreciated. We hypothesized that image-enhanced endoscopy (IEE) could improve object/lesion visibility regardless of CVD and tested this hypothesis with volunteers of varying color vision characteristics.

**Methods:**

Sixty objects/lesions (20 each of submucosal vessels during colorectal endoscopic submucosal dissection, superficial esophageal cancer, and early gastric cancer) were evaluated. Images with WLI and IEE (red dichromatic imaging [RDI], narrow-band imaging [NBI], or indigo carmine) were assessed for each object/lesion. Twenty evaluators (9 with general color vision: Type C and 11 with two types of CVD [7 with protanopia: Type P and 4 with deuteranopia: Type D]) scored vessel and lesion visibility using a four-level scale.

**Results:**

RDI significantly improved the visibility of submucosal vessels in Types P and D ( *P* < 0.0001) compared with WLI, but not in Type C. NBI significantly improved visibility of superficial esophageal cancer compared with WLI in all color vision types ( *P* < 0.0001). Indigo carmine significantly improved visibility of early gastric cancer compared with WLI in all color vision types ( *P* < 0.0001). Although visibility scores under WLI were significantly higher in Type C compared with Types P and D for all objects/lesions, intergroup differences appeared smaller under IEE due to improved visibility in Types P and D.

**Conclusions:**

IEE improves visibility of objects/lesions where color information plays a role in detection, regardless of CVD.

## Introduction

Color vision deficiency (CVD) is a condition in which distinguishing between certain colors becomes challenging. CVD affects approximately 8.0% of males and 0.4% of females 1 . This difference in prevalence between sexes occurs because the most common forms of congenital CVD are inherited in an X-linked recessive pattern 2 . Depending on the affected cone cells, CVD is classified into protanopia, deuteranopia, and tritanopia.

In many countries, color vision tests are not required for medical licensure 3 , so some endoscopists may have CVD without being aware. Research conducted in the Netherlands showed that 8% of gastrointestinal endoscopists were diagnosed with CVD and that CVD does not affect accuracy of colorectal polyp diagnosis 4 . However, the study could not strictly assess the impact of CVD on endoscopic diagnosis because a polyp is elevated from the surrounding mucosa. Visibility of endoscopic images can make a difference in diagnostic performance of lesions 5 . Therefore, considering that color tone is one of the most important pieces of information in gastrointestinal endoscopy and that a certain percentage of endoscopists have CVD from an epidemiological perspective, understanding the impact of CVD on endoscopic care and devising remedial measures, if necessary, would be beneficial for best practice.

Our previous study using computer simulation demonstrated that visibility of vessels during colorectal endoscopic submucosal dissection (ESD), which was poorer in protanopia (Type P) and deuteranopia (Type D) than in Type C (general color characteristic) under white-light imaging (WLI), was significantly improved using red dichromatic imaging (RDI) 6 7 . Based on these results, we hypothesize that use of Image-enhanced endoscopy (IEE) improves lesion detection regardless of color vision characteristics.

Meanwhile, because our previous study was based on computer simulations, we believe that assessment by actual human subjects is essential for evaluating object visibility under endoscopy because visibility may not be determined solely by color differences simulated in silico.

Beyond RDI, several other forms of IEE, such as narrow-band imaging (NBI) 5 and dye-based indigo carmine chromoendoscopy 8 , are widely used in daily practice. These forms of IEE significantly contribute to detection and qualitative diagnosis of superficial esophageal cancer and early gastric cancer 5 8 . To investigate the impact of these forms of IEE on endoscopic visibility, we designed a clinical study involving volunteers with various color vision characteristics.

In this study, considering the IEE methods used in daily practice, we employed RDI for submucosal vessels during colorectal ESD, NBI for superficial esophageal cancer, and indigo carmine for early gastric cancer as IEE images. We selected images in which color differences, rather than surface asperity, play a key role in recognizing objects during endoscopic procedures. To test our hypothesis that IEE improves visibility across a range of color vision characteristics, we compared visibility of lesions/sites in WLI versus IEE images for each participant.

## Patients and methods

### Study design

This cross-sectional observational study was conducted at Kyorin University Hospital. Images from upper and lower gastrointestinal endoscopies performed at Kyorin University Hospital between April 1, 2017 and August 32, 2023 were used, with all personal medical information removed. Colorectal ESD, superficial esophageal cancer, and early gastric cancer were imaged using WLI and RDI modes, WLI and NBI, and WLI and indigo carmine chromoendoscopy modes, respectively. For each lesion/site, WLI and IEE images were captured within the same field of view (one set). This study included 20 cases of colorectal ESD, 20 of esophageal lesions, and 20 of gastric lesions, totaling 60 sets of images. Characteristics of the esophageal lesions and gastric lesions are shown in Table 1 .

**Table:** **Table 1** Characteristics of esophageal and gastric lesions.

**Esophageal cancer**					**Gastric cancer**				
**Case**	**Location**	**Size (mm)**	**Color (under WLI)**	**Depth**	**Case**	**Location**	**Size (mm)**	**Color (under WLI)**	**Depth**
1	Mt	20	Redness	MM	1	M	17	Normal	T1a
2	Mt	20	Redness	LPM	2	M	40	Normal	T1a
3	Mt	15	Redness	LPM	3	L	15	Normal	T1a
4	Lt	4	Redness	EP	4	L	15	Redness	T1a
5	Mt	5	Redness	LPM	5	L	20	Redness	T1a
6	Mt	10	Redness	LPM	6	M	10	Redness	T1a
7	Lt	10	Redness	LPM	7	L	9	Redness	T1a
8	Mt	30	Redness	LPM	8	L	17	Redness	T1a
9	Mt	23	Redness	LPM	9	L	25	Normal	T1a
10	Mt	38	Redness	LPM	10	L	22	Normal	T1a
11	Mt	13	Redness	LPM	11	U	20	Redness	T1a
12	Ut	18	Redness	LPM	12	L	18	Pale	T1a
13	Lt	20	Redness	LPM	13	U	14	Redness	T1a
14	Mt	34	Redness	LPM	14	L	13	Redness	T1a
15	Ut	10	Redness	LPM	15	L	32	Redness	T1a
16	Mt	25	Redness	LPM	16	L	20	Pale	T1a
17	Mt	8	Redness	LPM	17	M	32	Redness	T1a
18	Mt	18	Redness	LPM	18	L	23	Normal	T1a
19	Mt	10	Redness	LPM	19	U	30	Redness	T1a
20	Mt	8	Redness	LPM	20	L	22	Normal	T1a
EP, epithelium; L, lower body; LPM, lamina propria mucosae; Lt, lower thoracicesophagus; M, middle body; Mt, middle thoracic esophagus; U,upper body; Ut, upper thoracal esophagus; WLI, white-light imaging.									

**Fig. 1:**
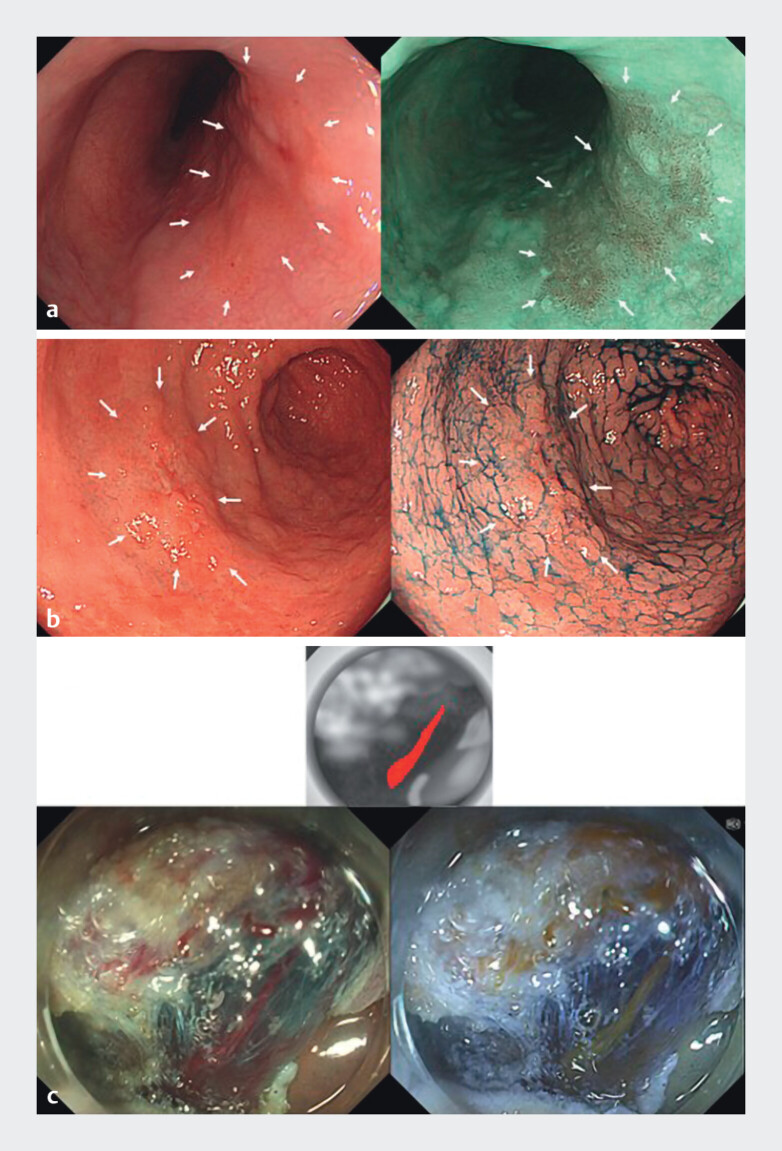
Representative evaluation images. For **a** superficial esophageal cancer and **b** early gastric cancer, a single lesion was displayed on the screen. The white-light image (WLI) was shown on the left and the image-enhanced endoscopy (IEE) image on the right, both captured within the same field of view. White arrows were used to mark the lesion in both images for identification. **c** For a blood vessel in the submucosa during colorectal ESD, a schematic diagram was additionally presented above the WLI and IEE images. This diagram featured a black-and-white background with the target vessel highlighted in red, ensuring that individuals with all color vision types could recognize the vessel being evaluated.

Colorectal ESD was performed using the EVIS X1 endoscopic system with a PCF-H290TI endoscope (Olympus, Tokyo, Japan). Images of superficial esophageal cancer and early gastric cancer were taken using the EVIS ELITE endoscope system equipped with either the GIF-H260 or GIF-H290. Intraoperative images of colorectal ESD contained at least one blood vessel that the operator needed to identify within the submucosa. In addition, each image depicted a single lesion of superficial esophageal cancer or early gastric cancer. To facilitate evaluation by participants without endoscopic knowledge, cancers and blood vessels under assessment were marked with arrows and schematic diagrams in each image ( Fig. 1 ).

### Visibility evaluation

**Fig. 2:**
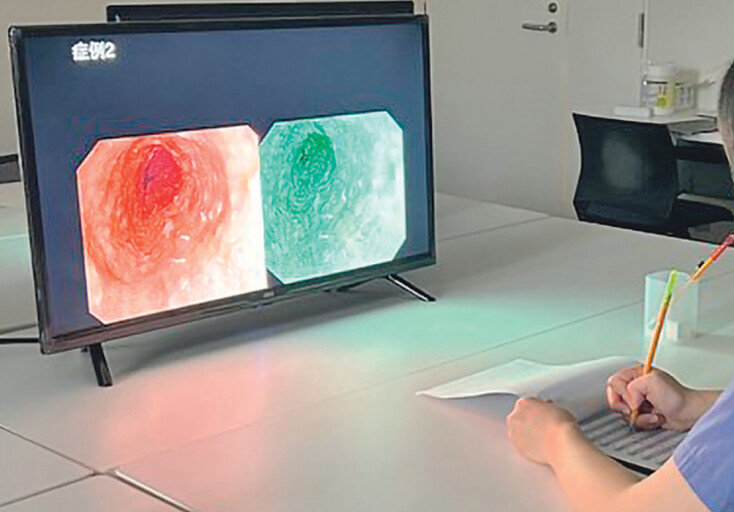
Study environment for assessing endoscopic visibility. A 32-inch monitor (3840 × 2160 resolution) was used, positioned 100 cm away from the participant. The room’s darkness was adjusted to mimic the conditions of an endoscopic examination room, and the monitor brightness was standardized at 10 to 15 lux.

Twenty participants, including individuals with CVD and those with general color vision, were recruited in cooperation with The Color Universal Design Organization CUDO ( https://cudo.jp ) following an ethically approved procedure. CUDO, a Japanese nonprofit organization, was established in 2004 to contribute to the realization of a society where all individuals can live more equitable and cultured lives by improving real-world color environments to accommodate diverse color vision characteristics. All participants had already been assessed and informed of their color vision characteristics (Type C, P, or D) by the organization using the Ishihara method or other methods and had previously participated in other CUDO activities promoting color universal design. Participants were selected on a first-come, first-served basis: nine for Type C, seven for Type P, and four for Type D ( Table 2 ). None of the participants were healthcare providers. These 20 evaluators assessed the visibility of two images per displayed set using a four-level score (1 indicating no visibility and 4 indicating the best visibility), as previously described in a study by Iwata and Kitamoto 9 10 investigating color visibility among different individuals ( Table 3 ). Each image set was displayed for 30 seconds, with a 5-second screen blackout between evaluation images. Images were presented in the following order: esophagus, stomach, and large intestine, arranged chronologically based on date of the endoscopic examination. To ensure an evaluation environment similar to that of regular endoscopic examinations in a clinical setting, a 32-inch FFF-LD32P5 monitor (resolution: 3840 × 2160) was used, with the viewing distance standardized at 100 cm. Screen brightness was also measured with a digital lux meter and standardized to 10 to 15 lux ( Fig. 2 ).

**Table:** **Table 2** Characteristics of participants.

**Color vision type**	**Type C** **(general color characteristic)**	**Type P** **(protanopia)**	**Type D** **(deuteranopia)**
Number of participants	9	7	4
Age, years	60 (40–70)	55 (45–69)	63 (55–70)
Sex, male/female	8/1	7/0	4/0
Data are presented as n or median (range).			

**Table:** **Table 3** Visibility scores.

**Visibility scores**	
**1**	I cannot see any lesions at all
**2**	I can see some of the lesions, but they are difficult to discern
**3**	I can see the lesions to some extent, so I would consider using this
**4**	The lesions are very easy to see, so I would definitely prefer to use this

### Outcomes

The primary outcome was to assess the difference in visibility scores between WLI and IEE images within each color vision type group (C, P, and D). The secondary outcome was to compare visibility of WLI and IEE across different color vision type groups.

### Statistical analysis

The Wilcoxon matched-pairs signed-rank test was used to compare visibility scores between WLI and IEE within each color vision type group (Type C, P, and D). The Kruskal–Wallis test followed by Dunn’s multiple comparisons test was used to compare visibility among the three color vision groups in WLI or IEE. Statistical significance was set at *P* < 0.05. All statistical analyses were performed using Prism version 9.5.1 (GraphPad Software, Boston, Massachusetts, United States).

### Ethics statement

This study was performed in accordance with the guidelines of the Declaration of Helsinki and was approved by the Institutional Ethics Committee of Kyorin University School of Medicine (Institutional Review Board approval No. 2277). The evaluators participating in the study were provided with a consent and explanation document approved by the ethical review committee. The study was thoroughly explained to them beforehand, and voluntary written consent was obtained. In addition, an opt-out option was provided for patients whose endoscopic images were used.

## Results

### Impact of IEE on visibility in each color vision type

Visibility of submucosal vessels, superficial esophageal cancer, and early gastric cancer under WLI and IEE was compared to determine whether IEE enhances visibility of lesions or objects where color information is a key factor for detection in each color vision type.

#### Submucosal blood vessel

**Fig. 3:**
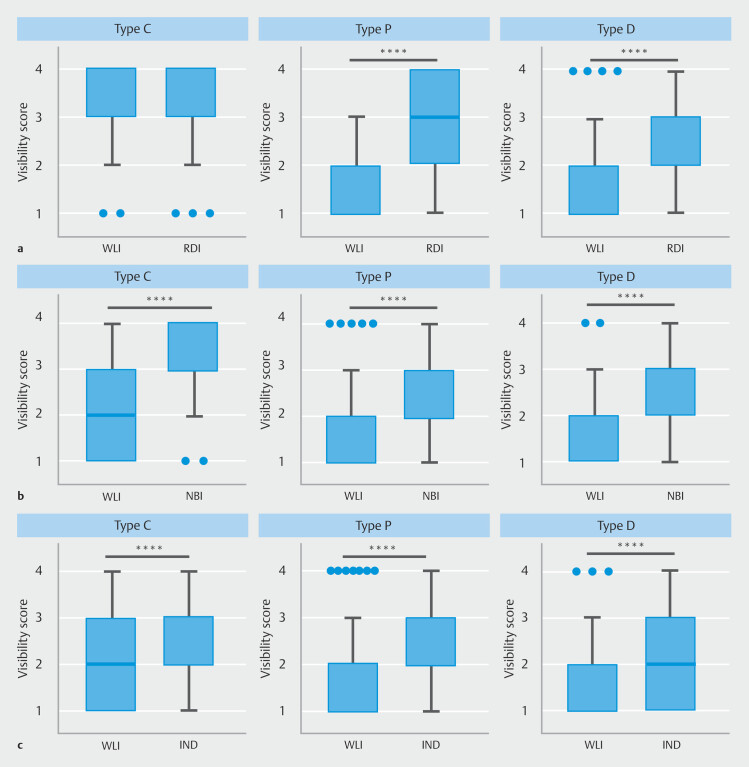
Impact of image-enhanced endoscopy on visibility of colorectal submucosal vessel, superficial esophageal cancer, and early gastric cancer. **a** Visibility scores for colorectal submucosal vessels under white-light imaging (WLI) versus red dichromatic imaging (RDI) in individuals with color vision Types C, P, and D. **b** Visibility scores for superficial esophageal cancer under WLI versus narrow-band imaging (NBI) in individuals with color vision Types C, P, and D. **c** Visibility scores for early gastric cancer under WLI versus indigo carmine in individuals with color vision Types C, P, and D. ****P < 0.0001, Wilcoxon matched-pairs signed-rank test.

In the Type C group, the visibility score for a blood vessel in colorectal ESD was 3.37 ± 0.05 (mean ± SEM) under WLI and 3.30 ± 0.06 under RDI. There was no significant difference between WLI and RDI images ( *p* = 0.29) ( Fig. 3 **a** ). In the Type P group, the visibility score was 1.34 ± 0.04 under WLI and 2.85 ± 0.09 under RDI ( *P* < 0.0001) ( Fig. 3 **a** ). In the Type D group, the visibility score was 1.79 ± 0.09 under WLI and 2.86 ± 0.09 under RDI ( *P* < 0.0001) ( Fig. 3 **a** ). RDI significantly improved visibility of submucosal blood vessels in Types P and D, whereas no significant effect was observed in Type C.

#### Superficial esophageal cancer

In the Type C group, the visibility score for superficial esophageal cancer was 2.05 ± 0.06 under WLI and 3.19 ± 0.06 under NBI ( *P* < 0.0001) ( Fig. 3 **b** ). In the Type P group, the visibility score was 1.67 ± 0.07 under WLI and 2.40 ± 0.08 under NBI ( *P* < 0.0001) ( Fig. 3 **b** ). In the Type D group, the visibility score was 1.71 ± 0.09 under WLI and 2.61 ± 0.11 under NBI ( *P* < 0.0001) ( **Fig. 3b** ). NBI significantly improved visibility of superficial esophageal cancer compared with WLI across all color vision types.

#### Early gastric cancer

In the Type C group, the visibility score for early gastric cancer was 2.15 ± 0.07 under WLI and 2.56 ± 0.08 under indigo carmine ( *P* < 0.0001) ( Fig. 3 **c** ). In the Type P group, the visibility score was 1.80 ± 0.07 under WLI and 2.51 ± 0.09 under indigo carmine ( *P* < 0.0001) ( Fig. 3 **c** ). In the Type D group, the visibility score was 1.70 ± 0.10 under WLI and 2.11 ± 0.12 under indigo carmine ( *P* < 0.0001) ( Fig. 3 **c** ). Indigo carmine chromoendoscopy significantly improved visibility of early gastric cancer compared with WLI across all color vision types.

### Influence of color vision types on endoscopic visibility

Based on our finding that IEE improved visibility of lesions and objects overall, regardless of color vision type, we next compared visibility scores under IEE among different color vision types.

#### Submucosal blood vessel

**Fig. 4:**
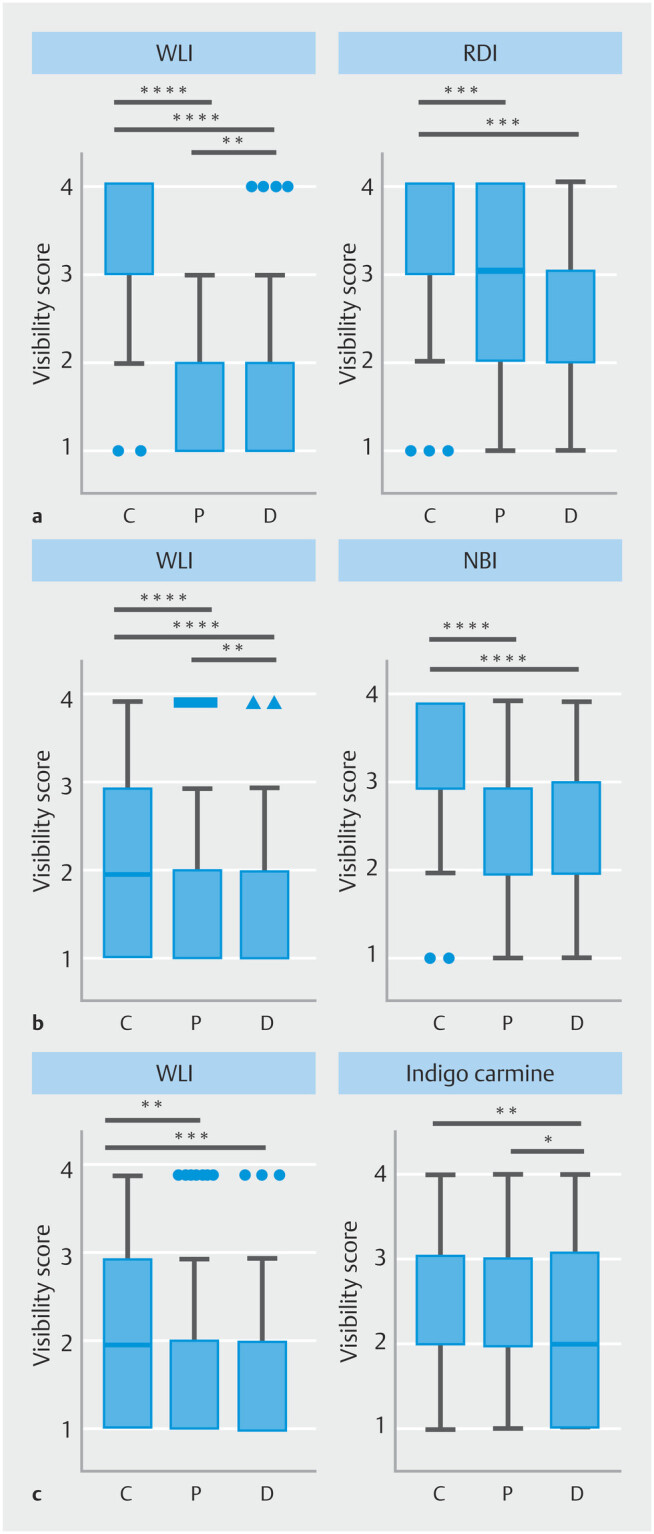
Influence of color vision characteristics on visibility of colorectal submucosal vessel superficial esophageal cancer, and early gastric cancer under WLI and IEE. **a** Visibility scores for a colorectal submucosal vessel under under white-light imaging (WLI) and red dichromatic imaging (RDI) comparing individuals with color vision Types C, P, and D. **b** Visibility scores for superficial esophageal cancer under WLI and narrow-band imaging (NBI) comparing individuals with color vision Types C, P, and D. **c** Visibility scores for early gastric cancer under indigo carmine comparing individuals with color vision Types C, P, and D. *P < 0.05, **P < 0.01, ***P < 0.001, ****P < 0.0001, Kruskal–Wallis test with Dunn’s multiple-comparisons test.

Visibility scores for Types C, P, and D were 3.37 ± 0.05, 1.34 ± 0.04, and 1.79 ± 0.09, respectively, under WLI. The scores for Type C were significantly higher than those for Types P and D ( *P* < 0.0001 for both comparisons), and the scores for Type D were also significantly higher than that for Type P ( *P* < 0.01). Visibility scores for Types C, P, and D were 3.30 ± 0.06, 2.85 ± 0.09, and 2.86 ± 0.09, respectively, under RDI. Although the scores in Type C were significantly higher than those in Types P and D ( *P* < 0.001 for both comparisons), the median visibility score across all color vision types was 3 ( Fig. 4 **a** ).

#### Superficial esophageal cancer

Visibility scores for Types C, P, and D were 2.05 ± 0.06, 1.67 ± 0.07, and 1.71 ± 0.09, respectively, under WLI. The visibility score for Type C was significantly higher than those for Types P and D ( *P* < 0.0001 and *P* < 0.001, respectively), whereas there was no significant difference between Types P and D. Visibility scores for Types C, P, and D were 3.19 ± 0.06, 2.40 ± 0.08, and 2.61 ± 0.11, respectively, under NBI. Although the median visibility score in each color vision type was 2 or higher, scores for Type C remained significantly higher than those in Types P and D under NBI ( *P* < 0.0001 for both comparisons) ( Fig. 4 **b** ).

#### Early gastric cancer

Visibility scores for Types C, P, and D were 2.15 ± 0.07, 1.80 ± 0.07, and 1.70 ± 0.10, respectively, under WLI. The visibility score for Type C was significantly higher than those for Types P and D ( *P* < 0.01 and *P* < 0.001, respectively). No significant difference was observed between Types P and D. Visibility scores for Types C, P, and D were 2.56 ± 0.08, 2.51 ± 0.09, and 2.11 ± 0.12, respectively, under indigo carmine. There was no significant difference between Type C and Type P, whereas scores for Type C ( *P* < 0.01) and Type P ( *P* < 0.05) were significantly higher than those for Type D. However, the median visibility score was 2 or higher across all color vision types ( Fig. 4 **c** ).

## Discussion

Compared with WLI, NBI improved visibility of superficial esophageal cancer regardless of color vision characteristics. This finding indicates that NBI is more effective than WLI for detecting superficial esophageal cancer across all color vision types. Similarly, visibility of early gastric cancer was enhanced with indigo carmine compared with WLI in all color vision groups.

Superficial esophageal cancer and early gastric cancer often present as red lesions due to irregular vessels, with the contrast between lesion redness and the surrounding normal mucosa being key for detection under WLI. However, individuals with Type P perceive redness as darker than those with Type C, and dark red appears almost black to them. Therefore, we hypothesize that they may struggle to detect lesions because the entire endoscopic field appears darker. Individuals with Type D have difficulty distinguishing nuances in red, which can make it challenging to differentiate lesions from the surrounding normal mucosa.

NBI utilizes two extremely narrow wavelengths of light. The narrower wavelength band likely contributed to improved visibility for individuals across all color vision types in this study. However, under NBI, lesions are identified based on the contrast between green (normal mucosa) and dark brown (cancer). Given our findings that Type C retained higher visibility scores than Types P and D when NBI was used, we speculate that this color combination is difficult to distinguish for individuals with Types P and D, which is consistent with a previous report 11 .

Indigo carmine chromoendoscopy enhances mucosal surface structure by accumulating blue dye in depressed areas, emphasizing irregularities in the tissue. This enhancement of surface structure benefits individuals of all color vision types. For individuals with Type P, red lesions appear darker, which increases contrast and improves lesion visibility. However, because individuals with Type D have difficulty perceiving subtle differences in red, color contrast is less useful to them than to those with Type C and Type P. Instead, it can be speculated they rely more on surface structure information. This may explain why visibility improved for individuals with Type D when using indigo carmine but remained lower than that for individuals with Type C and Type P.

Regarding visibility of blood vessels running in the submucosal layer during colorectal ESD, RDI significantly improved blood vessel visibility for Types P and D, although improvement in visibility scores was not apparent in Type C. We speculated that this was because the baseline score (3.37 ± 0.05 out of 4) was already too high to achieve statistical significance. Moreover, although previous studies have reported that RDI improves submucosal vascular visibility 12 13 , these studies did not mention whether the participants had CVD; therefore, presence of individuals with CVD cannot be excluded. In addition, the evaluators in those studies were endoscopists, whereas our study involved non-healthcare providers as evaluators. These differences in participant backgrounds may explain the discrepancy in RDI visibility scores, particularly in the Type C group. Overall, the findings for this study were consistent with our previous results using computer simulations of CVD 6 . Our prior study demonstrated that RDI has the potential to enhance the color difference between blood vessels and submucosal tissue, regardless of color vision characteristics, suggesting an improvement in blood vessel visibility across all color vision types. RDI employs red, amber, and green light with distinct characteristics to create contrast in deep tissue, rendering submucosal blood vessels in orange. Because orange is complementary to the deep blue submucosa in the background, this contrast remains largely unaffected by CVD, thereby improving blood vessel visibility.

Medical color vision testing is not currently required for medical licensing in many countries 3 . Although prevalence of CVD is higher in males than in females 10 , a considerable number of endoscopists may have CVD. Assessing an endoscopist’s aptitude based solely on their color vision characteristics is not appropriate; instead, it is essential to acknowledge the diversity in color vision among endoscopists and develop systems that ensure the safety of all endoscopic procedures by providing visual information in an accessible manner. In this study, a Type C participant remarked, “I could easily see the submucosal blood vessels under WLI in colorectal ESD images because I know that blood vessels are red.” However, this perception of “red” is challenging for individuals with Type P and Type D, highlighting the fact that “standard” images and atlases related to gastrointestinal endoscopy have been created predominantly by individuals with Type C. Given that a substantial number of endoscopists have CVD, incorporating the principles of universal color design in educational materials—such as endoscopy atlases and videos—represents a crucial unmet need for improving patient care quality in clinical settings. Ron Mace, a leading proponent of universal design 14 , defines it as “the design of products and environments to be usable by as many people as possible without the need for special adaptation or design.” We believe these principles should be embraced within the field of endoscopy. Although this study did not identify specific factors influencing visibility among individuals with different types of color vision, clarifying such factors could help determine which IEE modes are most effective for particular CVD subtypes.

This study has some limitations. First, because of the limited number of volunteers with CVD, it was not feasible to strictly match ages across groups, although the differences were not large. In addition, we could not assess and match the degrees of CVD among participants with Types P and D. However, this is the first study to evaluate visibility of endoscopic images among individuals with various color vision types, including CVD, providing significant insights into the impact of CVD on endoscopic visibility with WLI and IEEs. Second, we used endoscopic images from cases performed at a single institution, which may have influenced our findings. Although our facility follows a standardized setting for endoscopic procedures, different brightness settings or other parameters when recording images could potentially improve visibility for individuals with Types P and D. Furthermore, the concentration of indigo carmine used in endoscopy varies between facilities (we use a concentration of 0.07% to 0.10%, whereas some facilities may use lower concentrations), and these differences could affect visibility. Finally, this study focused on color vision and endoscopic visibility using images of superficial esophageal cancer, early gastric cancer, and submucosal blood vessels during colorectal ESD. However, in clinical practice, endoscopists rely not only on color but also on other visual cues such as surface irregularities and texture differences. Experienced endoscopists develop learning effects that enhance their recognition of lesions, but this study did not assess such effects because none of the participants were healthcare providers. Further studies incorporating higher brain functions will be necessary to understand how individuals with various color vision characteristics recognize lesions during endoscopy.

## Conclusions

This study demonstrated that IEEs improved the visibility of objects, such as cancers and blood vessels, across all color vision characteristics, including CVD.
